# The Calmodulin/Striatin Interaction Is Enhanced in Diabetic Hearts and Defines Novel Signaling Clusters Implicated in Cardiac Remodeling

**DOI:** 10.1021/acsomega.5c13122

**Published:** 2026-05-18

**Authors:** Stephanie Chacar, Cynthia Al Hageh, Liaqat Ali, Pierre Zalloua, M-Saadeh Suleiman, Frank Christopher Howarth, Ali A. Khraibi, Moni Nader

**Affiliations:** a Department of Medical Sciences, College of Medicine and Health Sciences, Khalifa University of Science and Technology, Abu Dhabi, P.O. Box 127788, United Arab Emirates; b Department of Public Health and Epidemiology, College of Medicine and Health Sciences, Khalifa University of Science and Technology, Abu Dhabi, P.O. Box 127788, United Arab Emirates; c Core Technology Platforms, 549112 New York University Abu Dhabi, Abu Dhabi, P.O. Box 129188, United Arab Emirates; d Harvard T.H. Chan School of Public Health, Boston, Massachusetts 02115, United States; e Bristol Medical School (THS), 152331 University of Bristol, Bristol BS2 8HW, United Kingdom; f Department of Physiology, College of Medicine and Health Sciences, United Arab Emirates University, Al Ain, P.O. Box 15551, United Arab Emirates; g Department of Biomedical Engineering and Biotechnology, College of Medicine and Health Sciences, Khalifa University of Science and Technology, Abu Dhabi, P.O. Box 127788, United Arab Emirates; h Department of Physiological Sciences, College of Medicine, Alfaisal University, Riyadh 11533, P.O. Box 50927, Saudi Arabia

## Abstract

Diabetic cardiomyopathy (DbCM) impairs cardiac performance through complex mechanisms, which limits effective therapies. We investigated the protein networks of calmodulin (CaM), a striatin (STRN)-binding protein implicated in DbCM, and compared them with the STRN network from the same tissues. Using CaM as bait, we precipitated protein clusters from left ventricles (LVs) of diabetic rat hearts at 8 and 24 weeks post-streptozotocin (post-STZ). Diabetic rats exhibited pathological remodeling, evidenced by increased heart-to-body weight ratio, β-MHC protein, and ANF mRNA expression. Western blotting showed elevated STRN, but not PP2A-A or -C subunits, in chronic stages. Proteomic analysis at 24 weeks revealed 49 differentially interacting proteins (DIPs) with CaM: 18 enhanced and 31 diminished in diabetic LVs. Functional annotation highlighted the recruitment of proteins in metabolic pathways (fatty acid elongation and lipid metabolism) and PPAR signaling, alongside reduced interactions in lipid response, glucocorticoid response, calcium signaling, and amino acid biosynthesis. Comparative analysis of CaM and STRN interactomes converged to 231 proteins implicated mainly in metabolic processes. Out of these, three proteins (Hnrnpm, Acot7, and Gsta3) surfaced as regulators of metabolic, oxidative, and post-transcriptional remodeling. These findings reveal novel functional roles for the CaM/STRN complex in DbCM and identify a shared signaling hub that may guide therapeutic strategies for diabetes-associated cardiac deterioration.

## Graphical abstract

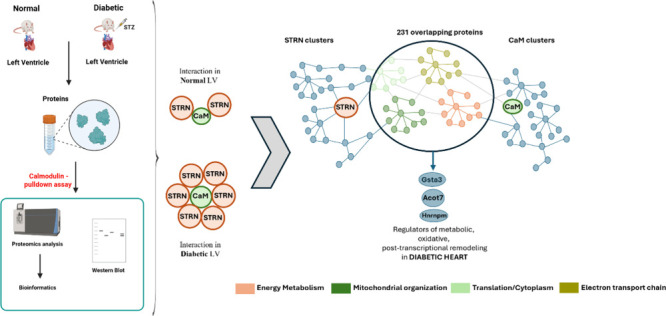

## Introduction

Diabetic cardiomyopathy (DbCM) is a pathophysiological condition in which heart failure occurs in patients with diabetes in the absence of coronary artery disease, hypertension, or valvular heart disease.^1^ There are several metabolic disturbances in type 1 diabetes (T1D) that contribute to the development of metabolic, structural, and functional abnormalities observed in the diabetic heart.^2^ There is currently no effective cure for DbCM due to the limited understanding of its molecular basis and the metabolic complexity of the diabetic heart.

We previously provided a comprehensive overview for a network of proteins that interact with striatin (STRN), a calmodulin (CaM) binding protein, in the diabetic heart, and we highlighted their functional implication in cardiometabolic disorders.^3,4^ CaM, a Ca^2+^ binding protein, is integral to the regulation and transduction of Ca^2+^ signaling in cardiomyocytes.^5^ With its pleiotropic role, CaM regulates a wide range of physiological processes by interacting with distinct target proteins across different cellular locations (i.e., the nucleus, the mitochondria, and the sarcoplasmic reticulum).^6–8^

On the other hand, several studies have highlighted a metabolic role for CaM. For example, CaM is essential for the Ca^2+^-dependent insulin secretion in pancreatic β-cells; overexpression of CaM impairs this process, indicating that regulated CaM levels are essential for proper β-cell function.^9,10^ In contrast, human umbilical vein endothelial cells exhibited reduced expression of CaM in diabetes, thus leading to the dysfunction of these cells and compromised angiogenesis.^11^ In the CaM-induced diabetic transgenic mouse model (OVE26), elevated CaM in pancreatic β-cells led to early-onset diabetes.^10^ Moreover, in the diabetic state, CaM engages in several interactions that impact cellular processes linked to diabetes-related dysfunction. For example, CaM binding to AS160, a vital regulator of glucose uptake, supports glucose absorption in response to muscle contraction, a crucial process for maintaining glucose homeostasis in diabetes.^12^ Also, CaM interacts with Rab3A, a protein involved in insulin secretion: disruptions of this interaction could affect insulin release, thus complicating diabetic metabolic control.^13^ With respect to its role in cardiometabolic disorders, the expression of CaM protein is reduced in diabetic cardiomyocyte, while exposure of these cells to insulin restores the expression of CaM.^14^ While CaM expression is reduced in diabetic hearts, the expression and activity of its target, (Ca^2+^/calmodulin-dependent protein kinase II) CaMKII, are upregulated, resulting in impaired Ca^2+^ handling, reduced heart performance, and cardiac hypertrophy.^15–17^ Although efforts have been made to target CaMKII in diabetic patients to alleviate the burden of DbCM, a therapy directed at CaM has not yet been developed, likely due to the incomplete understanding of its interactors and the complexity of CaM-regulated signaling pathways in the heart.^18,19^

Despite the implication of CaM in numerous metabolic processes, there is no information about the CaM protein clusters and their dynamics in hyperglycemia-induced stressed hearts. Given the importance of both STRN and CaM in cardiac metabolism, along with their binding capacity, we tailored our study to evaluate the CaM interactome and its dynamics in diabetic hearts, and to compare this interactome with that of STRN. We found a hub of differentially interacting proteins (DIPs) with CaM in diabetic LVs that is central for cellular pathways involved in pathological cardiac remodeling and dysfunction. Of interest, STRN showed increased interaction with CaM in diabetic hearts. The comparison of CaM interactors (2722 proteins) and STRN interactors (352 proteins) revealed a hub of 231 common proteins of which 3 proteins (hnrnpm, Acot7, and Gsta3) are mainly implicated in metabolic, oxidative, and post-transcriptional remodeling in DbCM. These findings describe novel functional signatures for CaM and STRN in the diabetic heart and pave the way for the identification of therapeutic targets for DbCM to reduce their cardiac dysfunction derivatives.

## Material and Methods

### Experimental Animals

The present study was approved by the Animal Research Oversight Committee (AROC) of Khalifa University (KU) of Science and Technology and by the Faculty of Medicine & Health Sciences Animal Ethics Committee of United Arab Emirates University (UAEU). The 8 and 24 week post-STZ (60 mg/kg body weight) rat models used here were previously characterized in our lab.^4,20^ In both models, animals remained hyperglycemic until sacrifice and showed increased heart weight to body weight (HW/BW) ratios consistent with pathological remodeling (cardiac hypertrophy). The number of animals used ranged from five to seven, depending on the experimental assay, as detailed in the following sections.

### Protein Extraction

Cardiac left ventricles were homogenized in an ice-cold lysis radioimmunoprecipitation assay buffer (RIPA buffer, cat. no. R0278, Sigma-Aldrich) supplemented with a complete protease inhibitor cocktail (cat. no. 11836145001, Roche) using an electric TissueRuptor (Qiagen). Samples were kept on ice for 30 min for a maximal yield of proteins. The homogenate was cleared by centrifugation at 10,000*g* for 5 min at 4 °C, and the supernatant was collected. Protein concentration of each sample was determined using the Pierce BCA protein assay kit (cat. no. 23225, Thermo Fisher Scientific).

### Calmodulin Pull-Down Assay and Western Blot

CaM Sepharose 4B beads (cat. no. 17-0529-01, GE Healthcare) were washed and equilibrated with RIPA buffer containing 2 mM CaCl_2_ and were incubated with 500 μg of proteins extracted from heart lysates from left ventricles (LVs) of control and diabetic rats overnight at 4 °C with constant shaking. Following overnight incubation, the protein complex was precipitated by a quick centrifugation at maximum speed, and the pelleted beads (containing the protein complex interacting with CaM) were subsequently washed, suspended in loading buffer (1×), and boiled for 5 min at 95 °C for protein denaturation and dissociation. The beads were centrifuged for 1 min at maximum speed, and the protein lysates (supernatant) were size-fractionated on a 10% polyacrylamide gel to evaluate the interaction status between CaM and target proteins, specifically STRN, PP2A-A, and PP2A-C. Proteins were then transferred to a polyvinylidene difluoride (PVDF) membrane (Bio-Rad Laboratories Inc., Irvine, CA, USA) under constant electrophoretic conditions (100 V, 400 mA, 2h). The PVDF membrane was blocked for 1 h at room temperature in TBS-Tween (TBST-T) blocking solution (Thermo Scientific, cat. no. 28360) and 5% nonfat dry milk (NFDM) and was subsequently probed overnight with gentle agitation at 4 °C with the appropriate antibodies in TBST-T and 5% NFDM: striatin (STRN) (cat. no. 610383, BD biosciences), PP2A-A subunit (cat. no. 2039, Cell Signaling Technology), PP2A-C subunit (cat. no. 2038 Cell Signaling Technology), and GAPDH (anti-glyceraldehyde 3-phosphate dehydrogenase, cat. no. 5174, Cell Signaling Technology) for loading control. The following day, the membrane was washed three times with TBS-T (5 min each) before incubation for 1 h at room temperature with specific antirabbit or antimouse secondary antibodies (Bio-Rad Laboratories). The protein bands were revealed with the ECL chemiluminescent substrate (Bio-Rad Laboratories, Inc.), and signals were detected using the Azure Biosystems 400 imaging system. Densitometry analysis of the protein bands was quantified using ImageJ (NIH). Statistical analyses were run using Graphpad Prism (version 10.0). The results were expressed as mean ± SEM of *n* samples. Unpaired *t* test was employed, and the results were considered statistically significant at *p* < 0.05.

### Cell Culture and AAV-Mediated STRN Overexpression

H9c2 cardiomyoblasts (cat. no. C0031002) were procured from AddexBio and handled according to the manufacturer’s protocol. Cells were cultured in Dulbecco’s modified Eagle’s medium (DMEM) (cat. no. 11995065, Gibco) supplemented with 10% heat-inactivated fetal bovine serum (FBS) (cat. no. 10082147, Gibco) and 1% penicillin–streptomycin (10,000 U/mL) (cat. no. 2321073, Gibco) in an incubator set to 37 °C and 5% CO_2_ atmosphere. H9c2 cardiomyoblasts were infected with custom-made adeno-associated viral particles (AAV), transducing Red Fluorescent Protein-STRN (RFP-STRN, Ad-mStrn-RFP, ADV-273389) or RFP (Ad-RFP, Ad-CMV-RFP 1660) alone as control (Vector Biolabs) at an MOI of 100. Overexpression efficiency was validated by Western blot assays.

### Proteomics Analysis

In addition to performing Western blot analysis on the CaM assay samples, we were also interested in assessing further potential interactions with CaM in our samples. The protein complex was labeled with TMT for quantitative proteomics analysis.

### Preparation of Protein Digest and High-pH Reversed-Phase Fractionation of Labeled Peptides

Eluted samples were reduced, S-alkylated, and digested from the beads (1.25 μg trypsin/lys-C, 37 °C, overnight). The peptide digests were TMT labeled and then pooled and desalted using a SepPak cartridge according to the manufacturer’s instructions (Waters). The eluted peptides from the cartridge were fractionated by a high-pH reversed-phase chromatography system (Ultimate 3000 HPLC, Thermo Fisher Scientific). In brief, the peptide digests were loaded onto a C18 Column (XBridge BEH, 130 Å, 3.5 μm, 2.1 × 150 mm, Waters) in buffer A and eluted with an increasing gradient of buffer B (20 mM ammonium hydroxide in acetonitrile, pH 10) from 0% to 95% over 60 min. The resulting fractions (eight in total) were dried and resuspended in 1% formic acid prior to online liquid chromatography–tandem mass spectrometry (LC–MS/MS, Orbitrap Fusion Lumos, Thermo Scientific).

### Liquid Chromatography–Tandem Mass Spectrometry

LC–MS/MS was performed on a fully automated Ultimate 3000 nano-LC system, in line with an Orbitrap Fusion Lumos mass spectrometer (Thermo Scientific). Mobile phases consisted of 0.1% formic acid for solvent A and 0.1% formic acid in 80% acetonitrile for solvent B. In brief, peptides in solvent A were injected onto a C18 nanotrap column (Acclaim PepMap, Thermo Scientific). After washing with 0.5% (v/v) acetonitrile and 0.1% (v/v) formic acid, peptides were resolved on a C18 analytical column (Acclaim PepMap, 250 mm × 75 μm, Thermo Scientific) over a 150 min linear gradient. The gradient was set as follows: 1–6% B for 1 min, 6–15% B for 58 min, 15–32% B for 58 min, and 32–40% B for 5 min. A 6 min wash at 90% B was used to prevent carryover, and 10 min equilibration at 1% B completed the gradient. A constant flow rate of 300 nL/min was used. Peptides were ionized by nanoelectrospray ionization at 2.0 kV using a stainless-steel emitter with an internal diameter of 30 μm (Thermo Scientific) and a capillary temperature of 300 °C.

The spectra were acquired with an Orbitrap Fusion Lumos system operated in data-dependent acquisition mode using an SPS-MS3 workflow. FTMS1 (Fourier transform mass spectrometry) spectra were collected at a resolution of 120,000, an automatic gain control (AGC) target of 200,000, and a max injection time of 50 ms. Precursors were filtered with an intensity threshold of 5000 according to the charge state (to include charge states 2–7) and with monoisotopic peak determination set to peptide. Previously interrogated precursors were excluded using a dynamic window (60 s, ±10 ppm). The MS2 precursors were isolated with a quadrupole isolation window of 0.7 *m*/*z*. ITMS2 (Ion Trap Mass Spectrometry) spectra were collected with an AGC target of 10,000, max injection time of 70 ms, and CID collision energy of 35%.

For FTMS3 analysis, the Orbitrap was operated at a resolution of 50,000, an AGC target of 50,000, and a max injection time of 105 ms. Precursors were fragmented by high-energy collision dissociation (HCD) at a normalized collision energy of 60% to ensure maximal TMT reporter ion yield. Synchronous precursor selection (SPS) was enabled to include up to 10 MS2 fragment ions in the FTMS3 scan.

### Data Curation and Statistical Analysis

The MS data were searched against the rat UniProt database protein groupings determined by Proteome Discoverer (PD) 2.4 and further refined using a custom script to select the best annotated master proteins. Peptide matches to multiple proteins were resolved by referring to the list of master proteins determined during the total protein analysis. Raw values representing the abundance of each protein were subjected to log transformation (log2). Only proteins with values in all five samples representing each condition were considered for the downstream analysis. Unsupervised and supervised statistical analyses were performed on CaM data that passed the quality control step. The analysis was performed using MetaboAnalyst v6.0.^16^

### Data Processing

Principal component analysis (PCA) and volcano plots were employed to explore the data. Raw data were processed to remove keratin and blood-related proteins (albumin and immunoglobulin). Data were preprocessed using standardization (*z*-score normalization) prior to PCA. The statistical software Python 3.11.6 was used for data analysis. The data set included five normal and five diabetic samples. PCA was applied to explore patterns of variation, while volcano plots combined log2 fold-change (FC) and −log10­(*p* value) from a two-tailed Student’s *t* test to assess differential protein expression between diabetics and controls. Differential protein expression analysis was conducted using an FC threshold of 1.3 and *p* value cutoff of 0.05. The top 10 upregulated and downregulated proteins were selected based on FC magnitude and statistical significance. *Z*-score normalization was applied to the expression values for heatmap visualization. Variable Importance in Projection (VIP) scores were calculated to identify key proteins contributing to the separation between cases and controls: VIP > 1, |FC| > 1.3, *p* value < 0.05.

### Pathway and Functional Enrichment Analysis

The DIPs (VIP > 1, *p* value < 0.05, and FC < −1.3 or FC > 1.3) were analyzed with STRING (STRING: functional protein association networks) for functional Gene Ontology (GO) enrichment analysis to identify biologically relevant pathways associated with the significantly more and less interacting proteins with CaM. The enrichment analysis included GO biological processes and KEGG pathways. Terms were selected based on the following thresholds: FDR ≤ 0.05, signal ≥ 0.01, strength ≥ 0.01, and minimum count of 2.

### Comparative Analysis of STRN and CaM Interactomes

To assess the overlap in protein interactions between STRN and CaM, we compared data sets obtained from two experimental approaches: a CaM affinity assay followed by proteomics analysis and a previously performed immunoprecipitation (IP) assay using STRN in normal and diabetic rat LVs. The protein accession numbers were processed and compared to identify shared and unique proteins among the two groups. A Venn diagram was generated to visualize the distribution and overlap of protein interactors between the CaM and STRN data sets. GO and pathway enrichment analyses were performed to identify biologically relevant pathways associated with the proteins at the intersection between IP and CaM assays. Biological process, molecular function, and cellular component categories were interrogated.

## Results

### The CaM/STRN Interaction Is Enhanced in Left Ventricles at Chronic Stages of Diabetes

While we previously reported that STRN and CaM interact in cardiomyocytes, the dynamics of this interaction was not evaluated in the pathologically remodeled heart in response to untreated diabetes. Therefore, we sought to evaluate the STRN/CaM interaction in a model of pathological cardiac remodeling at early and late stages of diabetes (8W vs 24W of diabetes-T1D). A CaM pull-down assay on protein lysates from LV of 8 and 24 week diabetic rats was performed followed by Western blot analysis to examine specific candidate proteins that typically interact with CaM, including STRN along with PP2A-A and -C subunits (all three proteins being part of the PP2A holoenzyme).^21^ Immunoblotting revealed that the association of CaM with STRN and PP2A-A and -C subunits was unchanged in the diabetic LV compared to age-matched normal rats at 8 weeks post-STZ (Figure 1A). Interestingly, the interaction between CaM and STRN was more pronounced in diabetic LVs compared to age-matched rats only at 24W post-STZ, while the CaM interaction with PP2A-A and -C subunits remained unchanged at both time points (Figure 1B). This is evident in the densitometry analysis of the protein bands presented as histograms for each CaM pull-down assay. These data suggest that the STRN/CaM interaction is independent of the PP2A-A and -C subunits, and its enhancement during the chronic stage of cardiac remodeling in diabetes highlights a stage-specific function in the diabetic heart.

**Figure 1:**
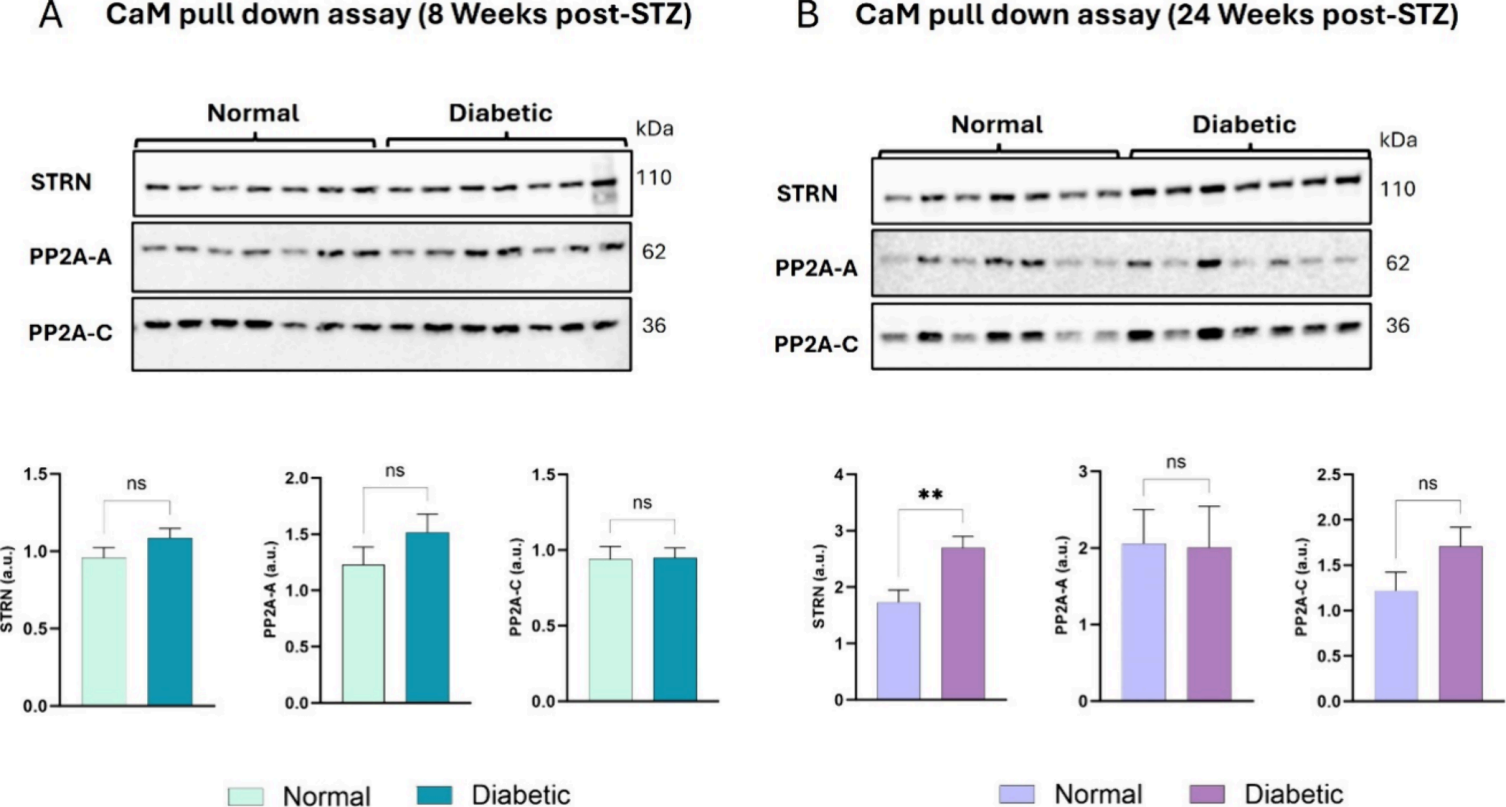
CaM pull-down assay and interaction patterns of STRN, PP2A-A, and -C subunits with CaM in diabetic left ventricles at early (8 weeks) and chronic (24 weeks) stages of diabetes. Western blot images and their corresponding histograms showing the interaction between CaM, STRN, and PP2A-A and -C subunits in normal and diabetic LVs at 8 weeks (A) and 24 Weeks (B) post-STZ. Values represent mean ± SEM (*n* = 7 samples per group for each protein); **p < 0.01.

### STRN Overexpression Does Not Alter the CaM/STRN Interaction in H9c2 Cells

To determine whether the increased CaM/STRN interaction observed in diabetic LVs is driven by elevated STRN expression in the diabetic heart^4^ or whether it is mediated by diabetes-related factors, we overexpressed RFP-tagged STRN (RFP-STRN) in H9c2 cardiomyoblasts and performed CaM pull-down assays. The CaM/STRN interaction profile was compared to that of cells transduced with the RFP control using both CaM pull-down and direct input protein assays across three independent experiments. Figure 2A shows an increase in the expression of the RFP-STRN cells compared to RFP, with a clear increase in the association between STRN and CaM in cells with higher expression of STRN. We further performed a ratio of the RFP-STRN/RFP bands and compared the increase in the STRN bands detected under both conditions (total proteins and CaM pull-down assay). We found no statistical significance between these two assays (Figure 2B), suggesting that an increase in the expression of STRN may translate into a promoted association between CaM and STRN irrespective of diabetes.

**Figure 2:**
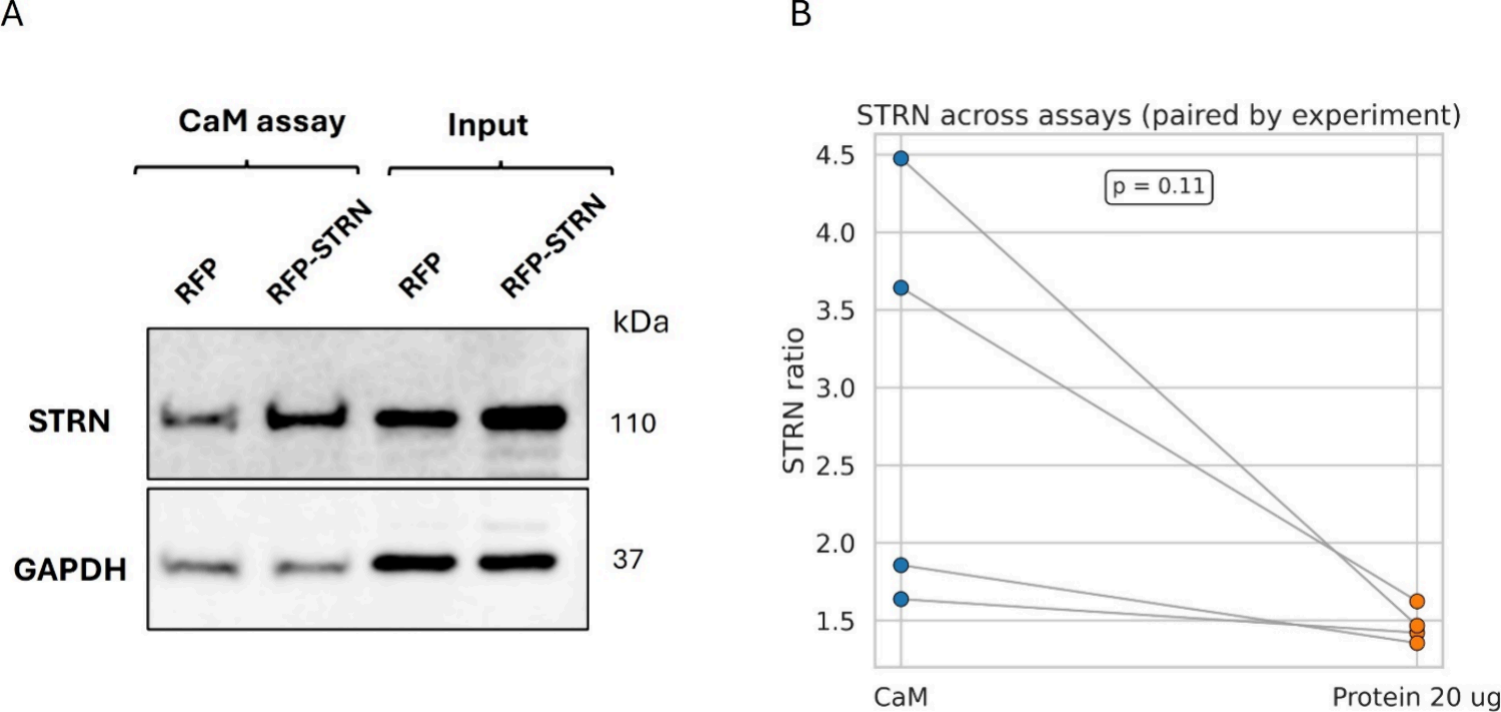
Effect of STRN overexpression on its association with CaM in H9c2 cells. (A) Representative Western blot showing STRN levels in CaM pull-down assays and corresponding input (protein lysates) from H9c2 cells transduced with RFP (control) or RFP-tagged STRN (RFP-STRN). GAPDH was used as a loading control for the input samples. (B) Quantification of STRN levels in CaM pull-down assays and input lysates across four independent experiments (*p* = 0.11, paired *t* test).

### Identification of Differentially Interacting Proteins with CaM in the Diabetic Left Ventricle

We previously reported that STRN interactors are uniquely shifted in the same diabetic hearts used for this study compared to the normal ones at 24 weeks of diabetes.^4^ Our findings herein showing an increase in the interaction between CaM and STRN at chronic stages of pathological remodeling prompted us to globally investigate the CaM/STRN-binding proteins in the diabetic LVs at this particular time point (24 weeks post-STZ). Therefore, we performed a proteomics analysis on the interactome released from the CaM pull-down assay to allow for a global exploration of CaM interactors in diabetic versus normal samples. The three-dimensional PCA plot in Figure 3A reveals the clear separation of the CaM interactors between normal and diabetic groups. The first three principal components account for 89.2% of the total variance in the data set, with PC1 explaining 75.1%, PC2 explaining 8.9%, and PC3 explaining 5.2% of the variance. This high percentage of variance indicates that PCA effectively captures the main patterns of variation in the protein expression data. The spatial distribution of samples shows distinct clustering of normal (blue) and diabetic (red) groups, suggesting significant differences in protein interaction with CaM between the two conditions.

**Figure 3:**
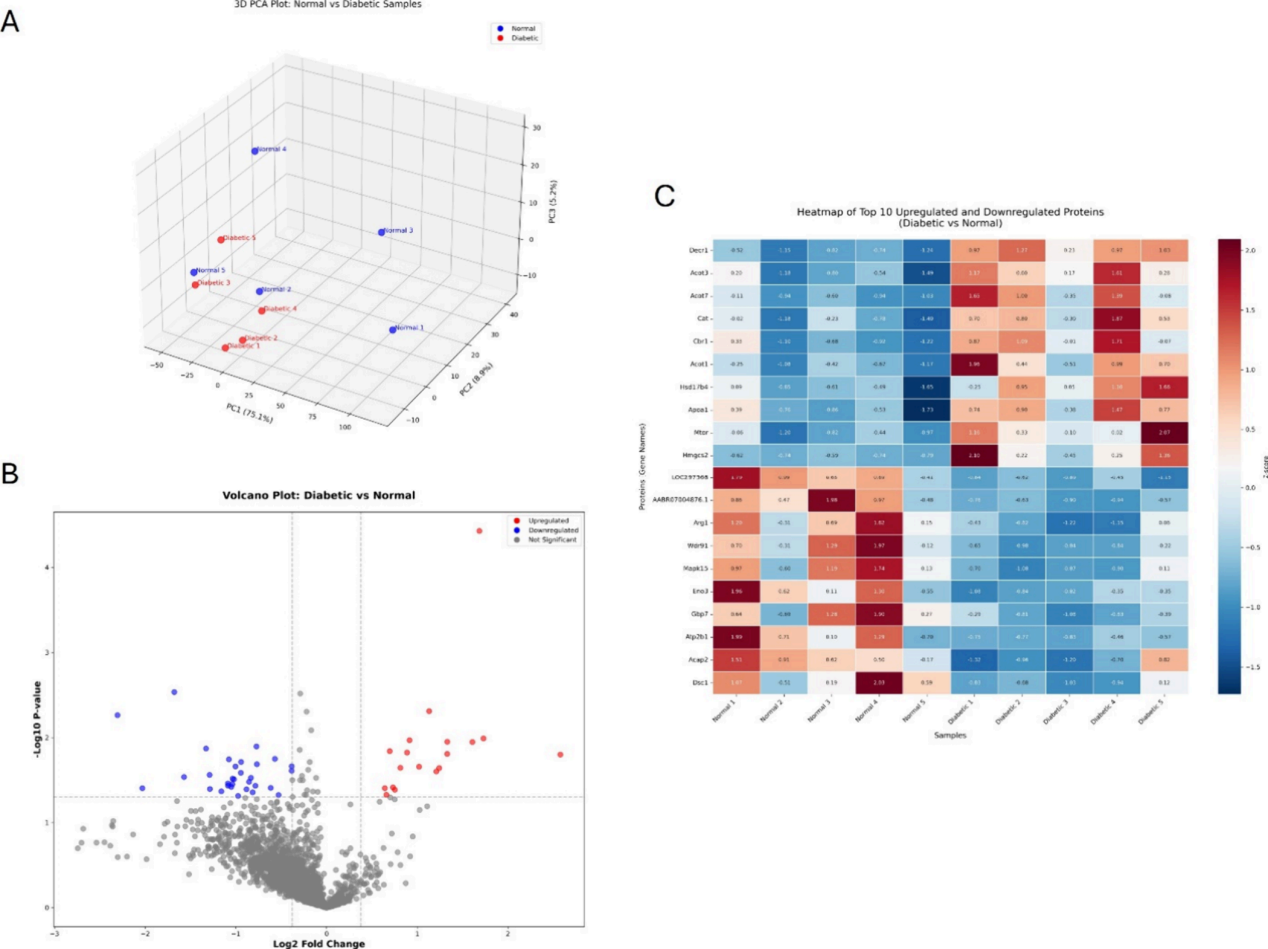
Proteomic profiling of CaM-interacting proteins in diabetic versus normal left ventricles. (A) Three-dimensional principal component analysis (PCA) plot of proteomics data from normal and diabetic samples. Blue dots represent normal samples (normal 1–5), and red dots represent diabetic samples (diabetic 1–5). Individual samples are labeled to show the distribution of biological replicates within each group. (B) Volcano plot shows differentially interacting CaM-binding proteins between diabetic and normal left ventricles. The results are presented in the upper part of the volcano plots, which represent the significance from the *t* test (−log10­(*p* value)) on the *y* axis and the log2 fold change (FC) on the *x* axis. (C) Heatmap visualization of the top 10 upregulated and top 10 downregulated CaM-interacting proteins in diabetic versus normal samples. *Z*-score normalized expression values are shown, with red indicating higher interaction and blue indicating lower interaction with CaM. Samples (normal 1–5 and diabetic 1–5) are presented in columns, and proteins (labeled by gene names) are in rows. Hierarchical clustering highlights distinct expression patterns between both groups.

The comparative analysis of these interactors between diabetic and normal conditions revealed significant alterations in protein abundance. The volcano plot in Figure 3B illustrates the distribution of proteins based on their FC (log2 FC) and statistical significance (−log10­(*p* value)), highlighting significantly downregulated (blue) and upregulated (red) proteins in diabetic compared to normal LV, with thresholds of *p* < 0.05 and |FC| > 1.3. This threshold yielded 49 DIPs with CaM. Among these DIPs, 18 proteins showed enhanced interaction (upregulated) and 31 exhibited reduced interaction (downregulated) with CaM in diabetic conditions compared to normal LV (Figure 3B and Table S1) **.**

The heatmap displays the top 10 upregulated and top 10 downregulated proteins in diabetic versus control LVs, revealing distinct DIP patterns between the two groups. Among the most highly interacting proteins, Hmgcs2 showed the highest fold change (log2 FC = 2.58, *p* = 1.59e-02) followed by Acot7 (log2 FC = 1.73, *p* = 1.03e-02) and Decr1 (log2 FC = 1.69, *p* = 3.75e-05). The most downregulated protein was AABR07004876.1 (log2 FC = −2.30, *p* = 5.47e-03) followed by Tfap2a (log2 FC = −2.03, *p* = 3.94e-02) and LOC297568 (log2 FC = −1.68, *p* = 2.93e-03) (Figure 3C).

### Proteomic Profiling Reveals Upregulated Calmodulin Interactors as Primary Drivers of Diabetic Heart Pathology

To identify key CaM interactors that may be driving the pathological remodeling in the diabetic heart, we performed a VIP score analysis on the identified DIPs. The VIP score ranking (Figure 4A) identified several proteins with high discriminatory power (VIP > 1), including Pdk4, Dbi, Acot1, Acot7, Gsta3, and Hnrnpm, suggesting a unique signature for CaM signaling in diabetic LV. A comparative plot showed that the mean VIP score of all upregulated proteins (2.495) was notably higher than that of all downregulated proteins (1.447), suggesting that upregulated proteins may play a more prominent role in defining the diabetic proteomic profile in the heart (Figure 4B).

**Figure 4:**
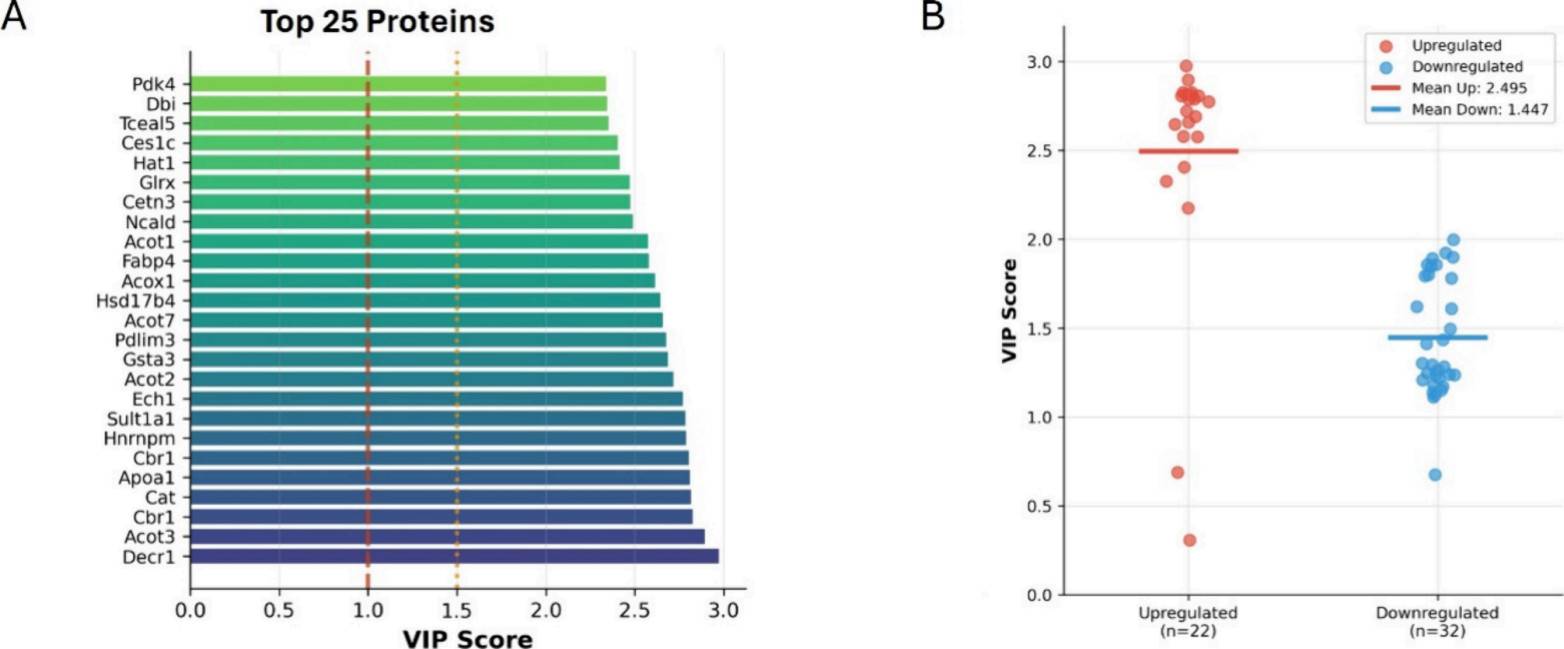
Variable Importance in Projection (VIP) analysis of differentially expressed CaM-interacting proteins between diabetic and control LV. (A) Bar plot showing the top 25 proteins ranked by VIP score, highlighting those with the highest discriminatory power between diabetic and control groups. (B) Scatter plot of VIP scores for upregulated (higher interaction with CaM; red) higher interaction with CaM and downregulated (lower interaction with CaM; blue) proteins. Each dot represents an individual protein, with horizontal lines indicating group means (upregulated: 2.49; downregulated: 1.44).

### Pathway and Functional Enrichment Analysis of Proteins with Altered Interaction with CaM in Diabetic Left Ventricles

To gain insight into the biological roles of the DIPs with CaM in diabetic LVs, we performed pathway enrichment analysis using STRING. Proteins that showed significantly increased interaction with CaM in diabetic LVs were enriched in several metabolic processes, including the fatty acid catabolic process, lipid metabolism, and acyl-CoA metabolic process (Figure 5A). The corresponding KEGG pathway analysis revealed enrichment in the PPAR signaling pathway, peroxisome-related functions, and fatty acid elongation (Figure 5B). In contrast, proteins with reduced interaction with CaM were enriched for GO terms related to cellular responses to lipids, glucocorticoids, and oxygen-containing compounds (Figure 5C). The KEGG pathway analysis of this group identified enrichment in calcium signaling and amino acid biosynthesis pathways (Figure 5D). Together, these data highlight that the CaM protein clusters reposition in the diabetic LVs to promote metabolic pathways and impede cellular responses to glucocorticoids and lipids and biosynthesis of amino acids.

**Figure 5:**
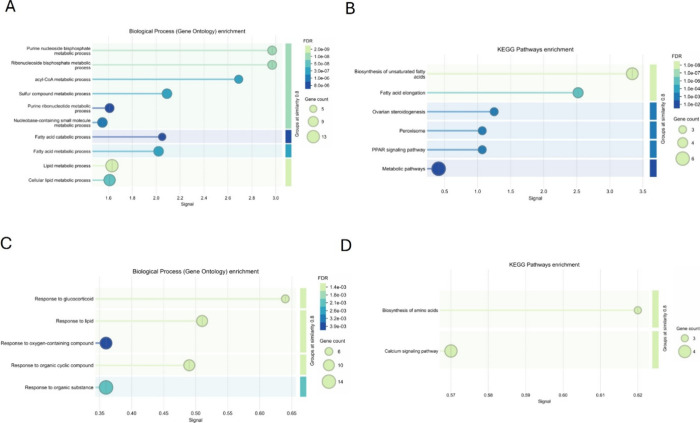
Functional enrichment analysis of differentially expressed CaM-interacting proteins in diabetic LVs. Enriched GO biological processes (A) and KEGG pathways (B) among proteins with an increased interaction with CaM in diabetic hearts. Top terms include lipid and fatty acid metabolism, acyl-CoA metabolic processes, and the PPAR signaling pathway. Enriched GO biological processes (C) and KEGG pathways (D) among proteins with reduced interaction with CaM. Notable terms include responses to lipids and glucocorticoids, along with enrichment in calcium signaling and amino acid biosynthesis pathways. The bubble size represents gene count, and the color indicates FDR-adjusted significance.

### CaM and STRN Co-interacting Proteins Define Mitochondrial and Contractile Networks in the Diabetic Heart

We previously reported an interaction between CaM and STRN in cardiomyocytes.^3^ We also reported a novel network of proteins (total of 352) interacting with STRN in rat LVs.^4^ Therefore, we ran a comprehensive analysis to compare the CaM interactome (2722 proteins) reported herein with our previously identified STRN interactors (proteins immunoprecipitated with STRN, both being assessed using the same rat cardiac tissues; diabetic and normal LV at 24 weeks post-STZ). The Venn diagram in Figure 6A illustrates the following distribution: 121 proteins were unique to the STRN IP group, while 2491 were exclusive to the CaM group, and 231 proteins overlapped between both data sets. Out of these 231 overlapping proteins, five showed significant changes in the interaction with CaM in diabetic LV: C3 (complement C3) exhibited reduced binding, while Ech1 (delta­(3,5)-delta­(2,4)-dienoyl-CoA isomerase), Gsta3 (glutathione *S*-transferase alpha-3), Hnrnpm (heterogeneous nuclear ribonucleoprotein M), and Acot7 (cytosolic acyl coenzyme A thioester hydrolase) showed increased interaction. This suggests a specific signature for the CaM/STRN protein cluster in diabetic LVs that may reflect a unique function in this setting.

**Figure 6:**
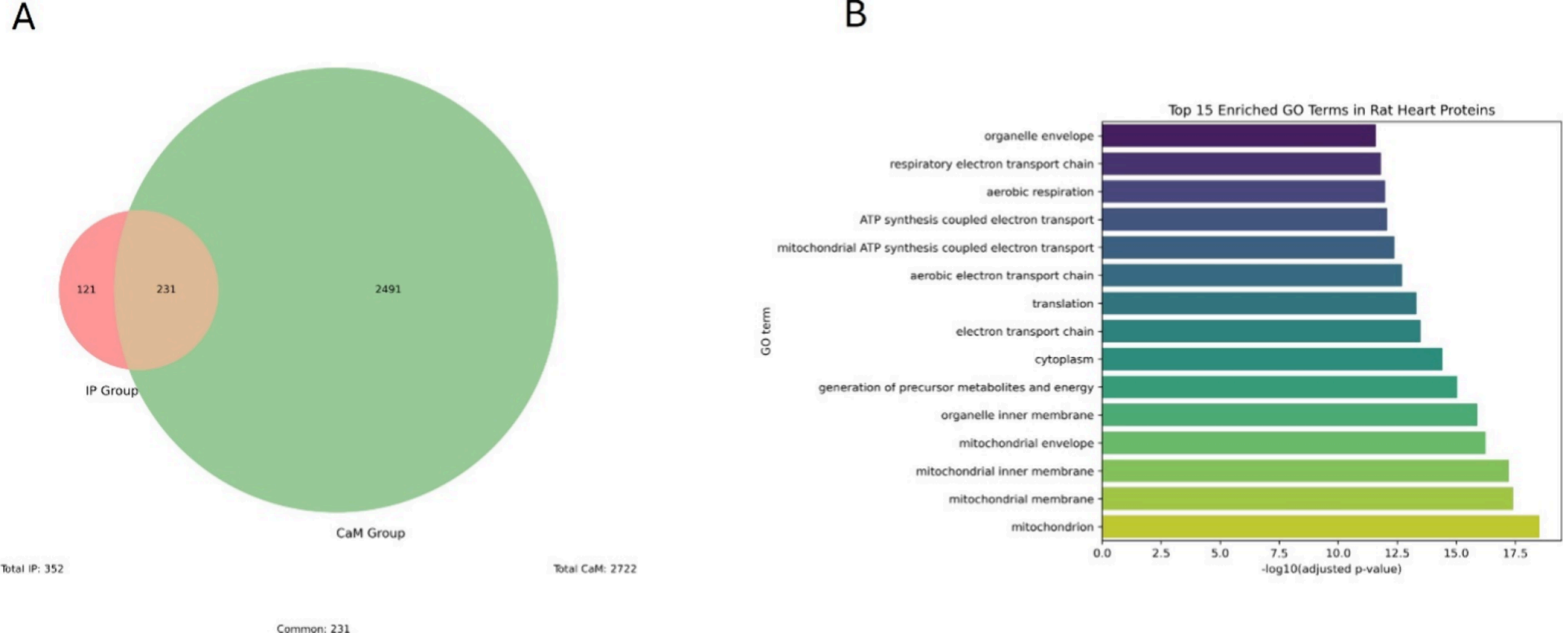
Comparison and functional profiling of CaM and STRN-IP interacting protein data sets. (A) Venn diagram showing the distribution of protein accessions between STRN IP group (left circle, red) and CaM group (right circle, green). The overlap represents 231 proteins common to both groups. (B) Bar graph representing the top 15 enriched GO terms among proteins overlapping between IP and CaM data sets. GO terms are shown on the *y* axis, and statistical significance is indicated by −log10­(adjusted *p* value) on the *x* axis.

To characterize biological and molecular pathways along with cellular components associated with the 231 overlapping proteins, we performed functional enrichment analysis. A total of 120 GO terms reached statistical significance (adjusted *p* value < 0.05). Enrichment was dominated by mitochondrial metabolic processes and contractile machinery components. Table S2 lists the top enriched terms, and Figure 6B illustrates their significance (−log10 adjusted *p* value). Together, these data identify a novel protein hub around the CaM/STRN complex that regulates important metabolic processes in the diabetic heart.

We further refined our analysis by searching for overlapping proteins between the proteins highly interacting with CaM (18 proteins) and those interacting with STRN under two different conditions: (1) proteomic data obtained from the IP of STRN from the diabetic LVs (247 proteins) in diabetic settings and (2) those interacting with STRN in diabetic and simultaneously normal and diabetic LVs (341 proteins). Both data sets were extracted from our previously published study.^4^ This integrative approach consistently identified three common proteins at the intersection of the CaM and STRN interactomes, namely, Acot7, Gsta3, and Hnrnpm (Figure 7A,B). These data suggest that these three proteins constitute a signaling hub for both CaM and STRN in the diabetic heart.

**Figure 7:**
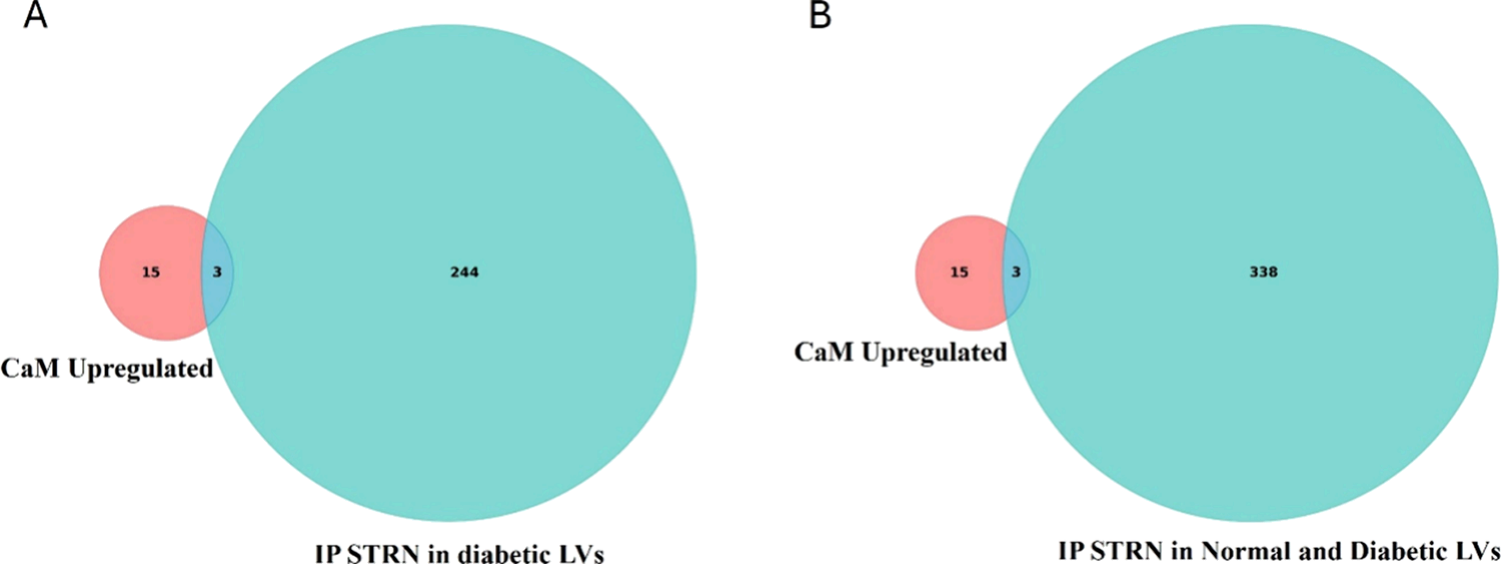
Comparative analysis of CaM upregulated interactors and STRN IP data sets. (A) Venn diagram comparing CaM upregulated proteins (left circle, red) with IP of STRN in diabetic LVs (right circle, green). The overlap represents three proteins common to both groups. (B) Venn diagram comparing CaM upregulated proteins (left circle, red) with IP of STRN in diabetic and simultaneously normal and diabetic LVs (right circle, green), showing the same three overlapping proteins.

Similarly, we checked for the common proteins at the intersection of the less interacting candidates with CaM (31 proteins) and (1) the STRN interactors from normal LVs (11 proteins) or (2) the STRN interactors from normal and simultaneously both normal and diabetic LVs (105 proteins). Interestingly, no overlapping proteins were identified in either comparison, suggesting that the cluster of proteins losing interaction with CaM under diabetic conditions is not linked to the STRN cluster, thus highlighting the importance of Acot7, Gsta3, and Hnrnpm signaling in this pathology (Figure S1).

## Discussion

The present study demonstrates, for the first time, that CaM forms a stronger complex with STRN in the diabetic LV during chronic remodeling. This interaction became pronounced 24 weeks after STZ-induced diabetes compared to 8 weeks, aligning with the transition from early metabolic disturbance to established DbCM. Enhanced CaM-STRN binding coincided with an adaptation of the CaM interactome, yielding 49 DIPs— 18 with increased interaction and 31 with reduced interaction with CaM—thereby revealing new metabolic and Ca^2+^-regulated signaling clusters pertinent to diabetic heart disease. Hnrnpm, Acot7, and Gsta3 emerged at the intersection of the DIPs highly interacting with CaM, and those interacting with STRN, in the diabetic LVs, portraying a novel signaling hub common to both CaM and STRN in diabetic hearts.

CaM serves as a central mediator linking diabetes to cardiac dysfunction through its regulation of Ca^2+^-dependent signaling pathways that become dysregulated in diabetic hearts. There is scarce information in the literature on the expression pattern of CaM in the diabetic heart; however, a couple of studies described a reduced expression of CaM in diabetic cells,^22^ namely, in myocytes.^14^ In fact, one of the roles of cardiac CaM is mediated by CaMKII. In the diabetic heart, chronic hyperglycemia and oxidative stress lead to enhanced CaMKII activation through methionine oxidation, thus promoting cardiomyocyte hypertrophy, apoptosis, and contractile dysfunction, all being characteristics of diabetic cardiomyopathy.^23,24^ CaM also modulates calcineurin activity, which drives pathological cardiac remodeling through NFAT-mediated transcriptional changes that contribute to hypertrophy and fibrosis in diabetic hearts.^25^

Of particular interest is the interaction between STRN and CaM under diabetic conditions. We previously reported the STRN/SLMAP interaction and the STRN/CaM protein complex in normal cardiomyocytes.^3,26^ Our data presented in Figures 1 and 2 indicate that the interaction between CaM and STRN is enhanced in the diabetic heart only at 24 weeks post-STZ (but not at 8 weeks post-STZ). This particular pattern was not seen in H9c2 cells overexpressing STRN (total STRN expression) when compared to the CaM-STRN interaction from the same cells *in vitro*. These imply that the enhanced interaction between CaM and STRN is most probably driven by adaptive cardiac remodeling at the chronic stage of DbCM (24 weeks post-STZ).^27^

While the proteomics analysis did not detect STRN as a prominent interactor with CaM (STRN peptides did not reach the detection threshold in LC–MS/MS), it identified other proteins involved in Ca^2+^ signaling and cellular stress responses aligning with the known Ca^2+^-dependent binding of the STRN family members to CaM. Notably, these included SLMAP, PP2A-C and CaMKII,^28–31^ although none reached statistical significance. Their role in DbCM, however, remains relevant; for instance, STRN anchors PP2A within the STRIPAK complex to regulate phosphorylation of CaM-sensitive targets.^28^ In DbCM, reduced PP2A activity combined with hyperactivated CaMKII creates an imbalanced phosphorylation environment that exacerbates calcium handling abnormalities, sarcoplasmic reticulum Ca^2+^ leak, and arrhythmogenesis, ultimately progressing to heart failure.^32^ Thus, the CaM/STRN axis represents a critical nexus where diabetic metabolic stress converges with Ca^2+^ signaling dysfunction to drive pathological remodeling and contractile failure.

In addition to these contentions, VIP score analysis yielded novel proteins that may serve as biomarkers for the diabetes-induced interaction with CaM in the myocardium under hyperglycemic conditions. The fact that the DIPs with enhanced interaction with CaM recorded a higher VIP score than those with reduced interaction with CaM suggests that these biomarkers are of functional importance in the settings of DbCM, most likely through the regulation of cardiac remodeling at this particular chronic pathological stage. Among these, Decr1 recorded the highest VIP score, underscoring its potential significance as a biomarker of diabetes-associated CaM interactions. In the diabetic heart, Decr1 expression and function are critical for maintaining mitochondrial fatty acid oxidation capacity, particularly as the diabetic heart becomes increasingly reliant on lipid substrates.^32,33^ Defects in β-oxidation enzymes can cause severe cardiomyopathy.^34^ Alterations in Decr1 activity contribute to incomplete fatty acid oxidation and the accumulation of partially oxidized lipid species, which impair mitochondrial function and promote oxidative stress.^35^ This mitochondrial dysfunction is a hallmark of DbCM and contributes to a reduced level of cardiac contractility, impaired relaxation, and eventual progression to heart failure.

Interestingly, the three dominant proteins at the intersection of CaM upregulated DIPs and STRN interactors, Hnrnpm, Acot7, and Gsta3, span distinct functional pathways including RNA processing, lipid metabolism via fatty-acid β-oxidation, and antioxidant defense. This convergence highlights metabolic alterations, oxidative stress regulation, and post-transcriptional control as connected features of DbCM,

The acyl-CoA thioesterase Acot7 plays a crucial role in regulating fatty acid oxidation by hydrolyzing medium- and long-chain acyl-CoA esters to free fatty acids and CoA. In DbCM, alterations in Acot7 expression and activity contribute to metabolic inflexibility, shifting substrate utilization toward fatty acids at the expense of glucose oxidation.^32,36^ This imbalance promotes lipotoxicity, impairs insulin signaling, and activates inflammatory pathways, driving cardiac remodeling and progression to heart failure.^37^ Notably, overexpression of fatty acid oxidation enzymes, including those regulated by Acot7, can reproduce key features of DbCM even in non-diabetic mice.^38^

In parallel, oxidative stress is a major driver of DbCM, and in a mouse model of Nrf2-driven reductive stress cardiomyopathy, Gsta3 was among the most enriched myocardial proteins, with transcriptomic data showing dose-dependent upregulation.^39,40^ It has been shown that in diabetic hearts, oxidative stress promotes mitochondrial dysfunction, impaired contractility, and pathological remodeling.^41^ In our diabetic LV model, Gsta3 showed increased interaction with CaM and emerged as a common interactor with STRN in diabetic LV, suggesting it may act as a redox-sensitive regulatory node within the CaM-STRN axis, thus influencing oxidative stress responses and structural remodeling.

Hnrnpm is an RNA-binding protein that regulates pre-mRNA splicing and processing across diverse cellular contexts^42,43^ and is critical for maintaining cardiac structural integrity and adaptive responses to stress. Recent evidence indicates a cardioprotective role for Hnrnpm, as circLRP6 was shown to protect cardiomyocytes from hypoxia-induced apoptosis through Hnrnpm-mediated upregulation of FGF-9.^44^ Other hnRNP family members, such as hnrnpu, are likewise essential for normal splicing in the heart, with their loss resulting in dilated cardiomyopathy in mice.^45^ Dysregulation of alternative splicing by RNA-binding proteins is increasingly recognized as a driver of cardiomyopathy and heart failure.^46^

## Conclusions

Collectively, these data position the CaM-STRN complex as a central scaffold that coordinates metabolic, oxidative, and gene-expression programs during diabetic remodeling. This opens several avenues for future investigations that could advance both mechanistic understanding and therapeutic development. First, the functional consequences of enhanced CaM-STRN interactions need validation through genetic and pharmacological approaches. Second, the temporal dynamics of the interactome reorganization between 8 and 24 weeks post-diabetes induction requires detailed characterization using longitudinal proteomic approaches. Third, how the CaM/STRN interactome unfolds to fuel the provoked signaling/functional cascades in late stages for DbCM remains to be determined.

## Limitations

Several limitations should be acknowledged in interpreting these findings. First, the sample size was modest, potentially underpowering the detection of low-abundance interactors. Second, the study employed a single diabetes model (STZ-induced), which may not fully recapitulate the heterogeneity of human diabetic cardiomyopathy. Third, an insulin-treated model would have provided further information on the dynamics of the CaM/STRN complex in the treated diabetic heart.

## Supplementary Material



## Data Availability

The mass spectrometry proteomics data have been deposited to the PRIDE archive (http://www.ebi.ac.uk/pride/archive/) via the PRIDE partner repository with the data set identifier PXD069737 and 10.6019/PXD069737. Username: reviewer_pxd069737@ebi.ac.uk. Password: PcJhZWSLK6em.

## Notes

Comparative Venn diagrams of CaM downregulated proteins and STRN interactors in normal and diabetic left ventricles (Figure S1); complete list of differentially interacting proteins identified in the diabetic heart (Table S1); and top enriched GO terms among proteins common to CaM pull-down and STRN IP data sets (Table S2) (PDF)
