# Supplementary material for: The Calmodulin/Striatin Interaction Is Enhanced in Diabetic Hearts and Defines Novel Signaling Clusters Implicated in Cardiac Remodeling
Source: ACS Omega. 2026 May 18;11(21):30801–11. doi: 10.1021/acsomega.5c13122 (PMC13234632; doi:10.1021/acsomega.5c13122)
Supplement: Supplementary file 1 [file ao5c13122_si_001.pdf]

The Calmodulin/Striatin Interaction is Enhanced in Diabetic Hearts and Defines Novel Signaling Clusters implicated in Cardiac Remodeling.

Stephanie Chacar[a], Cynthia Al Hageh[b], Liaqat Ali[c], Pierre Zalloua[b,d], M-Saadeh Suleiman[e], Frank Christopher Howarth[f], Ali A. Khraibi[g], and Moni Nader[a,h*].

[a] Department of Medical Sciences, College of Medicine and Health Sciences, Khalifa University of Science and Technology, Abu Dhabi, P.O. Box 127788, United Arab Emirates.

[b] Department of Public Health and Epidemiology, College of Medicine and Health Sciences, Khalifa University of Science and Technology, Abu Dhabi, P.O. Box 127788, United Arab Emirates.

[c] Core Technology Platforms, New York University Abu Dhabi, Abu Dhabi, P.O. Box 129188, United Arab Emirates

[d] Harvard T.H. Chan School of Public Health, Boston, Massachusetts, 02115, United States of America.

[e] Bristol Medical School (THS), University of Bristol, Bristol, BS2 8HW, United Kingdom.

[f] Department of Physiology, College of Medicine and Health Sciences, United Arab Emirates University, Al Ain, P.O. Box 15551, United Arab Emirates.

[g] Department of Biomedical Engineering and Biotechnology, College of Medicine and Health Sciences, Khalifa University of Science and Technology, Abu Dhabi, P.O. Box 127788, United Arab Emirates.

[h] Department of Physiological Sciences, College of Medicine, Alfaisal University, Riyadh, P.O. Box 50927, Saudi Arabia.

Address Correspondence to:

Moni Nader, PhD (*Email: mnader@alfaisal.edu and/or *E-mail: moni.nader@ku.ac.ae), Department of Medical Sciences, College of Medicine and Health Sciences, Khalifa University of Science and Technology, Abu Dhabi, United Arab Emirates and/or Department of Physiological Sciences, College of Medicine, Alfaisal University, Riyadh, Saudi Arabia.

Table of contents:

Figure S1: Comparative Venn diagrams of CaM downregulated proteins and STRN interactors in normal and diabetic left ventricles.

Table S1: Complete list of differentially interacting proteins identified in diabetic heart.

Table S2: Top enriched GO terms among proteins common to CaM pulldown and STRN IP datasets.

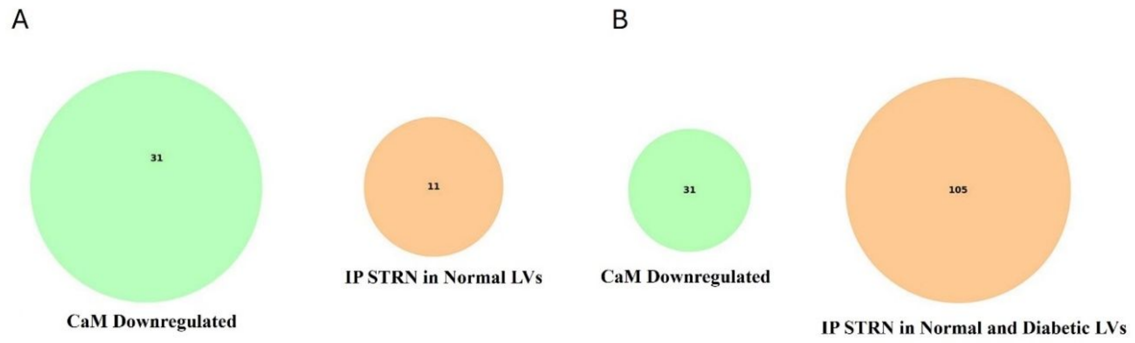

Figure S1: Comparative Venn diagrams of CaM downregulated proteins and STRN interactors in normal and diabetic left ventricles. A. Venn diagram comparing CaM downregulated proteins (Left circle-Green) with IP of STRN in Normal LV (Right circle-Orange), no overlap is observed. B. Venn diagram comparing CaM downregulated proteins (Left circle-Green) with IP of STRN in Normal and simultaneously Normal and Diabetic LV (Right circle-Orange), no overlap is observed.

Table S1: Complete list of differentially interacting proteins identified in diabetic heart. The table includes the log2 fold change (FC) and statistical significance (p-value).

Label	-Log10(P Value)	Log2 (Fold Change)	Regulation
Mybpc3	1.45	-1.04	Downregulated
C3	1.48	-0.85	Downregulated
Decr1	4.43	1.69	Upregulated
Eno3	1.74	-1.08	Downregulated
Acot2	1.64	1.24	Upregulated
LOC297568	2.53	-1.68	Downregulated
Cat	1.97	0.92	Upregulated
Hsd17b4	1.84	0.70	Upregulated

Ech1	1.64	0.81	Upregulated
Cbr1	1.66	1.02	Upregulated
Acot1	1.95	1.61	Upregulated
Cbr1	1.95	1.33	Upregulated
Apoa1	1.82	0.89	Upregulated
Fabp4	1.40	0.64	Upregulated
Acot3	2.31	1.13	Upregulated
Gsta3	1.39	0.75	Upregulated
Bcat2	1.54	-1.57	Downregulated
H1-5	1.40	-1.29	Downregulated
Trdn	1.46	-1.09	Downregulated
Acap2	1.66	-0.38	Downregulated
Thrap3	1.37	-1.16	Downregulated
Hnrnpm	1.41	0.73	Upregulated
Atp2b1	1.69	-0.77	Downregulated
Hmgcs2	1.80	2.58	Upregulated
Snrpd2	1.36	-0.81	Downregulated
Tfg	1.43	-0.78	Downregulated
Acot7	1.99	1.73	Upregulated
Mtor	1.81	1.33	Upregulated
Apoe	1.31	-0.98	Downregulated
Mtdh	1.56	-1.29	Downregulated
Ces1c	1.33	0.66	Upregulated
Adipoq	1.42	-1.05	Downregulated
Plpbp	1.39	-0.88	Downregulated
Ctsl	1.59	-0.94	Downregulated
Cetn1	1.53	-0.83	Downregulated
Kank1	1.51	-1.02	Downregulated
Snrpb	1.41	-0.62	Downregulated
ENSRNOG00000063333	1.32	-0.53	Downregulated
Arg1	1.89	-0.77	Downregulated
AABR07004876.1	2.26	-2.30	Downregulated
Gbp7	1.71	-0.94	Downregulated
Mapk15	1.75	-0.57	Downregulated
Rimoc1	1.61	-0.38	Downregulated
Sult1a1	1.60	1.21	Upregulated
P2rx3	1.43	-1.09	Downregulated
Wdr91	1.87	-1.33	Downregulated
Dsc1	1.66	-1.00	Downregulated
Tfap2a	1.40	-2.03	Downregulated
ENSRNOG00000062467	1.52	-1.04	Downregulated

Table S2: Top enriched GO terms among proteins common to CaM pulldown and STRN IP datasets. GO categories include Biological Processes (GO:BP) and Cellular Component (GO:CC), with significance assessed using FDR-adjusted p-values.

Ontology	GO term	Term size	Overlap	Adjusted p-value (FDR)
GO:CC	organelle envelope	620	31	2.50e-12
GO:BP	respiratory electron transport chain	87	15	1.55e-12
GO:BP	aerobic respiration	124	17	1.00e-12
GO:BP	ATP synthesis coupled electron transport	67	14	8.27e-13
GO:BP	mitochondrial ATP synthesis coupled electron transport	64	14	4.14e-13
GO:BP	aerobic electron transport chain	61	14	1.99e-13
GO:BP	translation	988	41	4.73e-14
GO:BP	electron transport chain	102	17	3.28e-14
GO:CC	cytoplasm	6764	116	3.80e-15
GO:BP	generation of precursor metabolites and energy	323	27	9.07e-16
GO:CC	organelle inner membrane	223	24	1.29e-16
GO:CC	mitochondrial envelope	358	29	5.74e-17
GO:CC	mitochondrial inner membrane	196	24	5.97e-18

GO:CC	mitochondrial membrane	325	29	3.94e-18
GO:CC	mitochondrion	1023	48	2.96e-19
